# Evaluation of Wait Times for Otolaryngology Appointments in Illinois

**DOI:** 10.1002/oto2.63

**Published:** 2023-07-12

**Authors:** Evan A. Patel, Trevor A. Poulson, Manushi Shah, Ashok A. Jagasia

**Affiliations:** ^1^ Department of Otolaryngology–Head and Neck Surgery Rush University Medical Center Chicago Illinois USA

**Keywords:** health care disparities, PS/QI

## Abstract

**Objective:**

The objective of this study was to quantify the wait times that patients may encounter for common clinical diagnoses when seeking otolaryngology care, while determining whether a wait time disparity exists based on geographic location within Illinois.

**Methods:**

In November 2022, a list of Illinois otolaryngologists was obtained from www.entnet.org. Using a uniform script, each of the 291 otolaryngologists were contacted. The caller posed as a new patient with either sudden sensorineural hearing loss, a neck mass, or chronic sinusitis. Each clinic was called 3 times and wait times were recorded. One hundred fifty‐eight otolaryngologists were included in the analysis.

**Results:**

The average statewide wait time for a new patient presenting with sudden unilateral hearing loss, a neck mass, and chronic sinusitis was 18.0, 22.6, and 25.5 days, respectively. There was no statistically significant difference between urban and rural wait times.

**Discussion:**

Although wait time differences were noted, the lack of urban versus rural *p* value significance may be attributed to the small sample size (*n* = 11) of rural otolaryngologists in Illinois. However, the overall wait times in this study were longer compared to those reported in other studies, suggesting that the current number of otolaryngologists in Illinois is inadequate to meet the public need.

**Implications for Practice:**

We have demonstrated that the current demand for otolaryngology care is outstripping the existing supply in Illinois. This suggests that an emphasis should be placed on training more otolaryngologists, or increasing the use of physician extenders, while incentivizing otolaryngologists to practice in rural areas.

**Level of Evidence:**

5

There has long been debate noted within academic literature regarding whether the supply of general otolaryngologists is able to adequately meet the demand for otolaryngological services across the United States. Although this issue of supply and demand is complex, it has significant consequences for the future of the field as well as the accessibility of care to patients. Too few otolaryngologists can decrease the accessibility of specialized head and neck care for patients and potentially impact the quality of care provided. However, an excess number of otolaryngologists could potentially result in underemployment and increased medical costs.^1^

Long waiting periods to obtain an appointment (national average wait time for a new patient of 13.2 days) may suggest an inadequate supply of otolaryngologists.^2^ This is also reflected by studies on physician extenders such as Nurse Practitioners and Physician Assistants. From 2012 to 2017, there was a 51% increase in the number of otolaryngology advanced practice providers (APPs) compared with a 2.2% increase in the number of physician providers.^3^ There is also an anticipated shortage of 1620 otolaryngology physicians by 2025 per projections from the US Department of Health and Human Services.^4^

Given that one of the most cited health care disparities in the United States is the inequality between urban and rural health care, encouraging providers to practice in these areas is crucial in bridging the gap. Rural Americans are on average more overweight, more likely to smoke tobacco, older, and have a higher prevalence of chronic diseases when compared to their urban counterparts.^5^, ^6^ A recent study found that 25% of rural households had a member who struggled to access care for a medical issue during the COVID‐19 pandemic, and nearly 17% of rural households had a member who was unable to receive a necessary surgical procedure.^7^ These factors are exacerbated by limited access to health care facilities, especially in the case of specialist care in fields such as otolaryngology. There is a notably smaller number of academy‐certified general otolaryngologists whose primary practice locations can be found in the rural counties of Illinois compared to urban locations.^8^ This results in a significant rural population with limited access to otolaryngologic care.^8^ To our knowledge, there is no existing literature related to the average wait times experienced by rural and urban patients seeking otolaryngology care in the United States.

In order to better analyze this situation, we first sought to determine the number of general otolaryngologists per capita in rural versus urban counties in Illinois. Then, by posing as a new patient, the appointment wait time for an emergent clinical issue (sudden hearing loss of the right ear), urgent clinical issue (lump in the neck), and less urgent issue (chronic sinusitis) were collected to serve as an indicator of patient access to otolaryngological services. The objective of this study was to better quantify the wait times that patients may face for common clinical diagnoses when seeking otolaryngology care, while determining whether there is a disparity in these wait times depending on the rurality of the clinician.

## Methods

**Figure 1:**
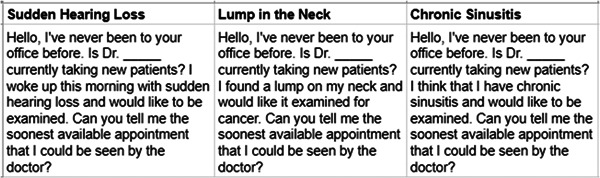
The uniform script that was outlined as part of the study methodology. Based on the diagnosis being examined, the caller would read the relevant script when contacting each office.

In November 2022, a list of 291 board‐certified otolaryngologists from Illinois was obtained from the American Academy of Otolaryngology (AAO) World Wide Web site which includes data on approximately 93% of otolaryngologists practicing in the United States.^9^ Using a uniform script (Figure 1), each of the 291 otolaryngologists in Illinois were called up to 3 times, with each call posing as a new patient with a sudden hearing loss, a lump in the neck, or chronic sinusitis to determine the wait time for each of these 3 maladies until the next available appointment. Physicians who did not see all 3 of these diagnoses (sudden hearing loss, neck mass, and chronic sinusitis) were excluded from the study. This group was primarily composed of fellowship‐trained physicians practicing only cosmetic, pediatric, or surgical otolaryngology by referral only. Specialists who were willing to schedule patients with all 3 diagnoses were included, regardless of their fellowship status or primary focus in practice. In addition, otolaryngologists who were not accepting new patients while on maternity leave or impending retirement, who were recently retired from clinical practice, or had moved from the state were excluded. Some offices required a form of insurance to be scheduled. These offices were excluded as well. The study population, therefore, included 158 practicing otolaryngologists in Illinois.

The caller informed the office administrative assistant that an appointment was being requested for the otolaryngologist and the patient was a new patient with sudden hearing loss. No name was provided. There was no reference to any insurance plan, and an appointment with a physician extender was refused if offered. The same office was called again twice more, once as a new patient with a lump in the neck, and again as a new patient with chronic sinusitis. All telephone calls were made between November 1 to November 30, 2022, and each of the 3 calls were made on different days but not longer than 5 business days apart. The order in which the calls were made was randomly assigned, as was the time of day the calls were placed. A letter of exemption for this study was obtained from the investigational review board of Rush University Medical Center in Chicago, Illinois.

Wait times were calculated as the number of calendar days from the date of the call until the date of the appointment offered. We established the average wait time in Illinois for a new patient with a diagnosis of sudden hearing loss, a lump in the neck, and chronic sinusitis, and examined the effect of population densities on the wait times. The classification of an urban or rural practice was determined using the zip code and county in which the practice operated. The definition of urban and rural was determined using the criteria used by the Illinois Department of Public Health, which states “Rural is defined as a county not part of a metropolitan statistical area (MSA), as defined by the U.S. Census Bureau; or a county that is part of an MSA but has a population fewer than 60,000.”^10^

All data were entered into a spreadsheet and analyzed using Pearson coefficient correlations with values for *p* less than 0.05 considered statistically significant. Statistical software was used to perform the statistical analyses that included examining any correlation between wait times, rural/urban classification of a geographic area, and the relationship between the number of otolaryngologists in an area and the population served. All statistical analysis was performed using GraphPad Prism version 8.0.0 for Windows (GraphPad Software, San Diego, www.graphpad.com).

## Results

**Figure 2:**
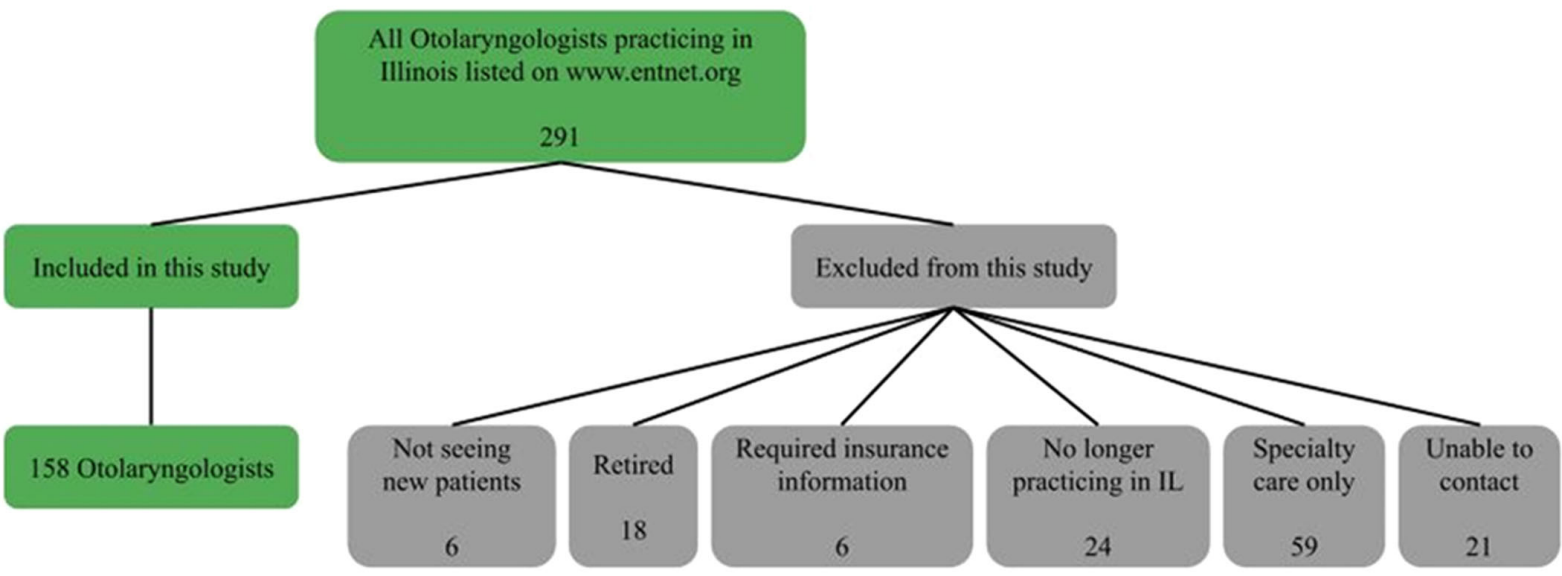
The breakdown of otolaryngologists listed on the American Academy of Otolaryngology website as practicing in the state of Illinois, and the exclusion criteria for otolaryngologists not included in our analysis.

Using the number of otolaryngologists registered with the AAO‐Head and Neck Surgery (HNS) with primary office locations within Illinois (291) as an estimate, along with the total population within Illinois (12,830,632), provides us with a total of 2.27 otolaryngologists/100,000 population.^11^ This number is a rough estimate, and the true value may be lower, given that a substantial number of the 291 otolaryngologists listed have retired or moved from the state (14.4%). Using 2020 US Census Bureau data, the number of otolaryngologists was found to increase with the population of Illinois counties (*r* = 0.9816, *p* < 0.0001) (Figure 2). Urban counties demonstrated a higher concentration, yielding an average of 2.43 otolaryngologists per 100,000, whereas in rural counties, there are only 0.99 otolaryngologists per 100,000 people.

**Figure 3:**
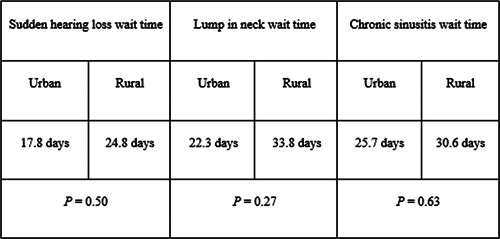
Average wait times found for sudden hearing loss, neck mass, and chronic sinusitis. The breakdown between urban and rural wait times for each of these diagnoses is shown as well.

Of the 291 otolaryngologists listed on the AAO World Wide Web site, only 158 were able to be successfully contacted and able to treat sudden hearing loss, neck mass, or chronic sinusitis. 133 otolaryngologists listed on the site were excluded from our analysis for various reasons (Figure 3).

**Figure 4:**
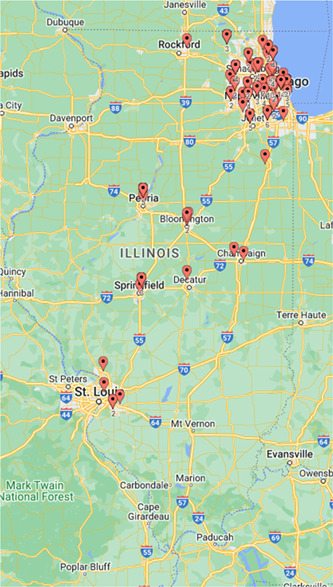
The geographic distribution of the 158 otolaryngologists that were included in this analysis throughout the state of Illinois.

The geographic distribution of the 158 otolaryngologists contacted in Illinois was plotted using online maps (Figure 4).^12^ Approximately 93% of contacted otolaryngologists practiced in urban counties, with over half (52.4%) of the 291 otolaryngologists having a primary practice location in Cook County alone. In addition, 96.6% of otolaryngologists specializing in certain procedures (i.e., Cochlear implantation), patient populations (i.e., pediatrics), or areas of focus (i.e., cosmetics) were located in urban counties within Illinois. We were able to identify only 20 of the 291 listed otolaryngologists as having a primary practice location in a rural county. Only 11 of the 158 otolaryngologists who were eligible for inclusion practiced in a rural county. The average total years in practice for the 147 urban otolaryngologists included in this analysis was 20.7 versus 21.9 years for the 11 rural otolaryngologists. However, 23.10% of urban otolaryngologists completed a fellowship versus only 9.00% of rural otolaryngologists.

**Figure 5:**
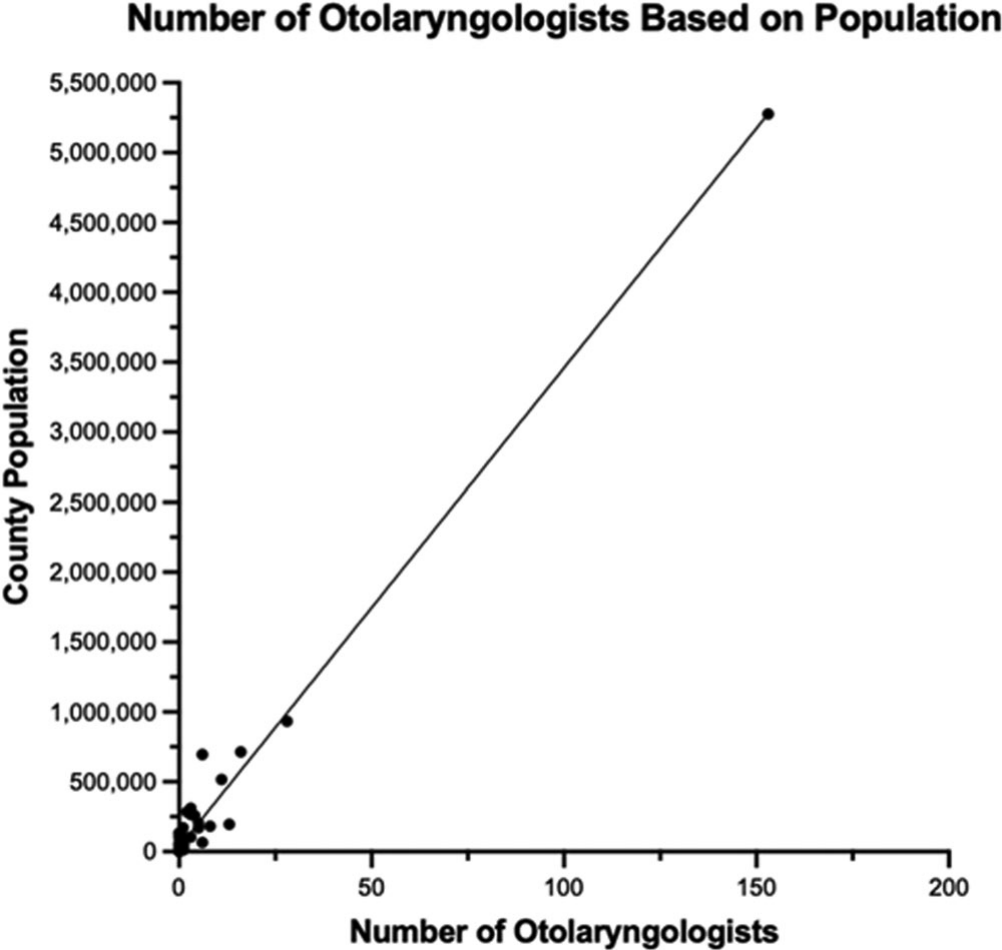
The relationship between the number of otolaryngologists within each county and the overall population of that county. County populations were determined using 2020 US Census data.

The average wait time for a new patient presenting with sudden hearing loss was 18.0 days. A new patient with a lump in the neck had a statewide average wait time of 22.6 days and a new patient presenting with chronic sinusitis had an average wait time of 25.5 days. The average wait time for each condition was calculated and tested for significance (Figure 5). The variation in wait times for new patients had a range of 0 to 122 days but was not directly correlated with the population of an area.

When compared against the urban sudden hearing loss wait times, both the urban lump in the neck and urban chronic sinusitis wait times yield significant *p* values of 0.039 and 0.001, respectively. There were no significant values (*p* < 0.05) between any of the rural wait time groups. Although there were no statistically significant p‐values when comparing urban versus rural wait times for each group, this can likely be attributed to the small sample size (*n* = 11) of rural otolaryngologists taking new patients in Illinois. The small sample size of rural physicians led to standard deviations of 33.3, 32.2, and 31.8 days for sudden hearing loss, lump in the neck, and chronic sinusitis wait times, respectively (Figure 5). The standard deviations of the rural wait times were more than 11 days greater than their respective counterparts in the urban wait time groups.

## Discussion

The wait times for an otolaryngology problem in this study was longer compared to wait times reported in other studies (average wait time of 13.2 days).^2^ These data suggest that the current number of otolaryngologists in Illinois (2.27 otolaryngologists /100,000 population) is inadequate to meet the public need.^4^, ^13^, ^14^ Problems caused by an undersupply of otolaryngologists are compounded when otolaryngologists are more accessible for patients in urban counties when compared to rural counties. Long wait times delay diagnosis by a matter of days but may have more profound effects if wait times discourage patients from seeing the otolaryngologist or direct them to other providers with less skill than a board‐certified otolaryngologist in making otolaryngology (ENT)‐related diagnoses.^14^

As regional anatomic specialist, otolaryngologists are well‐positioned to provide care to rural populations. The ability to offer both medical and surgical treatments while maintaining a breadth of understanding across multiple organ systems and disease processes allows otolaryngologists to treat patients of all ages. Furthermore, ENT complaints are among the most common reasons for rural patients to visit a physician.^15^ Many otolaryngologic diseases require prompt treatment and close surveillance, such as oral cancers. Despite the clear need for specialized ear, nose, and throat care in rural environments, the US otolaryngology workforce remains preferentially located in urban and suburban environments. Recent studies of midwestern^8^ and southeastern^15^ states identified significantly fewer per‐capita otolaryngologists in rural regions.

Of course, the tendency for otolaryngologists to be concentrated in heavily populated areas is not exclusive to Illinois. A recent AAO Practice Profile survey reported a mere 11.9% of otolaryngologists practicing in nonmetropolitan areas (defined here as a population of <50,000)^16^ and Vickery et al. identified 61.8% of US otolaryngologists practice in a metropolitan area with a population of at least 1 million, whereas these areas represent only 55.3% of the population.^17^ Gadkaree and colleagues evaluated the distribution of otolaryngologists at the county level and among hospital referral regions in 2020.^18^ Their findings indicated that rural counties had fewer otolaryngologists per population and, on multivariable analysis, county‐level otolaryngology density was positively associated with highest education quartile and highest income quartile.^18^ The concentration of otolaryngologists among high‐income urban areas further exacerbates health care disparities.^15^ Nationwide, there are significantly more practicing otolaryngologists per capita in urban areas compared to rural areas (*p* < 0.05),^18^ apart from West Virginia, where the difference was not statistically significant (*p* = 0.33).^18^

A notable finding was that the wait times were not directly correlated with the population of an area. One might expect a shorter wait time for heavily populated areas where there are more otolaryngologists per capita. However, this issue is multifactorial as the demand for elective, cosmetic, or specialized procedures may be greater in urban areas, and otolaryngologists in urban areas may devote more of their time to elective procedures. In addition, the coverage area of each otolaryngologist may extend well into rural areas based on the otolaryngologist reputation and services provided. Thus, in our highly mobile society, some patients from rural areas may favor traveling to cities to seek otolaryngologic care.

Other factors may explain the lack of correlation between wait time and the per capita distribution of otolaryngologists. Rural otolaryngologists may spend more time in the clinical practice of otolaryngology than their counterparts in the city. These findings may also be explained in part by the fact that women may make up a larger percentage of the otolaryngologist workforce in cities and work, on average, fewer hours per week than their male counterparts.^19^ The possibility also exists that more rural otolaryngologists are using physician extenders to keep the waiting time down.

This study suggests that in Illinois there may be less pressure to provide incentives to encourage otolaryngologists to practice in rural areas as the wait times are similar. If we agree that the average wait times are too long, however, we need increased numbers of medical otolaryngologists wherever they are distributed. This could be accomplished by increasing the number of otolaryngologists residents or encouraging otolaryngology residents to pursue careers in more generalized otolaryngology.

When compared to other specialties, the nationally reported average wait time for an otolaryngology appointment as a new patient (13.2 days) was shorter than for other specialties, such as pediatrics (17.1 days), cardiology (18.5 days), urology (19.2 days), and primary care (21.5 days).^2^ With regards to the state of Illinois, it appears that the wait times from this study (ranging from 18 to 25.5 days depending on diagnosis) were more in line with other specialties as opposed to the currently reported national otolaryngology wait time averages. However, these overall average numbers do not consider the potential differences and challenges that rural patients may face when it comes to accessing care. All of the aforementioned specialties have reported significant geographic disparities regarding access to care for rural populations, as a greater concentration of physicians’ favor practicing in urban areas, regardless of specialty.^20^, ^21^, ^22^, ^23^

Ultimately, as a growing body of evidence indicating a physician shortage builds, there will be increasing pressure for governmental funding of increased otolaryngology residency positions. New positions would be filled easily, as there is an excess of otolaryngology applicants each year. In 2022, 574 medical students applied to match at 1 of 361 otolaryngology residency positions.^24^ Between 2015 and 2020, the number of students applying increased by 9.3% each year, however, there was only a 3.6% increase annually in training positions.^25^ However, one alternative to increasing the number of residency positions may lie in the geographic distribution of practicing otolaryngologists. If there is a notable oversupply of otolaryngologists practicing in urban areas while rural populations remain underserved, it may be possible to direct government funding of positions to individuals who agree to practice in these regions.

Alternatively, we may need more physician extenders, a trend that is already occurring. From 2012 to 2017, there was a 51% increase in the number of otolaryngology APPs compared with a 2.2% increase in the number of physician providers.^12^, ^26^ The 2017 AAO‐HNS Socioeconomic Survey and AOA Practice Benchmarking Survey noted that 37% of polled practices were seeking to hire an otolaryngology nurse practitioner to help augment their practice.^16^ A majority of rural counties (72%) in 2017 reported zero otolaryngology providers, and a greater proportion of rural counties (5%) were served exclusively by APPs as compared with urban counties (3%).^3^ Otolaryngology APPs are more likely to practice in rural settings (14%) versus otolaryngology physicians (8%).^27^

There are several limitations of this study. One is that the insurance status was not taken into account, although previous studies have shown no significant difference in wait times for patients with private insurance companies versus Medicare.^28^ The collection of data only from the state of Illinois is an obvious limitation of this study as well. Illinois' ratio of 2.3 otolaryngologists per 100,000 people is slightly below the mean of 2.66 (SD 0.66) nationwide.^29^ A geographically large state with low population density such as Wyoming or Alaska might be expected to show significantly longer wait times for patients throughout the state and especially in rural/remote areas at great distances from major population centers. Another limitation of this study includes the low sample size of rural otolaryngologists within the state that were able to be contacted. With so few available physicians for data collection, it is possible that our statistical analysis does not capture the entire scope of the hurdles that rural populations must overcome to access equitable care.

## Implications for Practice

The diagnosis of sudden hearing loss is of particular concern because of the potentially permanent implications of a delay in diagnosis. In fact, the rate of hearing recovery following audiogram within the first few days of onset is 87%, with a week, 87%, 2 weeks 52%, and 10% or less after 3 months.^30^, ^31^, ^32^, ^33^, ^34^, ^35^, ^36^ Many of the office administrative assistants who were contacted as a part of this study appeared to be unaware of the urgency behind a sudden hearing loss diagnosis. Many of those who did urged us to visit the emergency room due to lack of scheduling availability, or attempted to schedule us with a physician extender.

To address the question of supply and demand in otolaryngology, our results demonstrate that the current demand for otolaryngology care may potentially be outstripping the existing supply in Illinois, which has a relatively average number of otolaryngologists per capita compared to national data. It was also observed that, although there is a disparity in the distribution of otolaryngologists across Illinois, the wait times for appointments were surprisingly uniform in urban and rural areas. However, the large concentration of physicians in urban counties across the state has left many large rural counties with no practicing otolaryngologists at all. While wait times in rural counties that had at least 1 otolaryngologist were not statistically significant in terms of different wait times when compared to urban counties, our data does not assess the wait time for a patient who has no nearby otolaryngologist, leaving large geographical swathes of rural‐dwelling Illinois citizens unaccounted for. This suggests that an emphasis should be placed on training more otolaryngologists, and/or increasing the use of physician extenders, while providing incentives for otolaryngologists to practice in underserved rural areas.

## Author Contributions

**Evan A. Patel**, conceived, drafted, edited, and approved final manuscript and takes full responsibility for its content; **Trevor A. Poulson**, conceived, drafted, edited, and approved final manuscript and takes full responsibility for its content; **Manushi Shah**, conceived, drafted, edited, and approved final manuscript and takes full responsibility for its content; **Ashok A. Jagasia**, conceived, drafted, edited, and approved the final manuscript and takes full responsibility for its content.

## Disclosures

### Competing interests

None.

### Funding source

None.
